# Diseases of Children

**Published:** 1892-02

**Authors:** E. Van Goidtsnoven

**Affiliations:** Atlanta, Ga.


### DISEASES OF CHILDREN.

BY DR. E. VAN GOIDTSNOVEN, ATLANTA, GA.

In my last paper, I treated of Cholera Infantum. Certain groups of symptoms are met with in the study and treatment of diseases of children, which often lead a physician astray, and cause him to mistake one disease for another. Anterior polio-myelitis acuta, commonly called infantile paralysis, is often encountered, and almost as often mistaken for cholera infantum.

Anterior polio-myelitis consists in primary atrophy of the nerve cells of the anterior horns of the spinal cord (motor tract), and in secondary complicating myelitis.

By far the largest number of patients are infants from one to three years of age. The disease often begins with indigestion, intestinal catarrh, or morbid dentition. During dentition the nervous system of a child is in a more irritable state, and, perhaps, more likely to suffer from injurious influences. This may explain the frequency with which infantile paralysis occurs during the first and second year, and why its incipiency may present some points of analogy with that of cholera infantum. Its progress, however, soon enables one to establish the differentiation: for it evolves into akinesis, wasting of muscular tissue, paralysis and deformities. I refer the reader to the masterly works of Charcot, Leguin, Hammond, etc., for further particulars of that disease, and simply mention it here, in order to caution the practitioner against the possibility of an erroneous diagnosis.

Cholera infantum is frequently followed by ileo-colitis, an inflammation of both the large and small intestines. This inflammation is confined mostly to the mucous membrane, the secretions of which are increased; and an increased peristaltic action having been set up, there is more or less diarrhœa. Oftener, however, ileo-colitis is a destructive disease from the beginning. Cold, irritating ingesta, or decomposition from the food and the production of irritant material from this, may likewise cause it. The appetite is impaired and if the child be nursing, the milk, having an acid smell, is thrown up by the stomach. The resulting diarrhœa is sometimes charged with bloody mucus, and is distinguished from dysentery by its feculence. When the stools contain much blood and mucus, the straining during each operation is invariably very severe. In the treatment of this disease, I have obtained most satisfactory results from the following prescription:

10. R. Hydrargyri chloridi mitis, gr. i.
Bismuthi Salicylatis,
Salolis,
Pulveris carbonis salicis ligni: aa gr xii.

Misce. Tere optime et in chartas duodecim (No. xii.) divide.
Signa. One (1) powder every four (4) hours.

I also insist upon the necessity of washing out the bowels antiseptically, and the prescription, No. 9, given in my last number, answers that purpose very satisfactorily.

